# Early Recurrence of Non-neuroendocrine Primary Cutaneous Mucinous Carcinoma of the Eyelid After Surgical Excision: A Case Report

**DOI:** 10.7759/cureus.115342

**Published:** 2026-08-28

**Authors:** Erika Ogi, Tomomi Kaiho, Ryusuke Hashimoto, Jun-ichiro Ikeda, Toshiyuki Oshitari, Jiro Yotsukura, Takayuki Baba

**Affiliations:** 1 Department of Ophthalmology and Visual Science, Chiba University Graduate School of Medicine, Chiba, JPN; 2 Department of Diagnostic Pathology, Chiba University Graduate School of Medicine, Chiba, JPN; 3 Department of Ophthalmology, International University of Health and Welfare Narita Hospital, Narita, JPN; 4 Department of Ophthalmology, Kakisu Eye Clinic, Togane, JPN

**Keywords:** eyelid neoplasm, local recurrence, negative surgical margins, neuroendocrine differentiation, non-neuroendocrine pcmc, primary cutaneous mucinous carcinoma

## Abstract

Primary cutaneous mucinous carcinoma (PCMC) is a rare malignant cutaneous adnexal neoplasm prone to local recurrence. Neuroendocrine differentiation has been recognized in a subset of these tumors, and emerging evidence suggests that its presence may be associated with differences in clinical behavior and prognosis. Reports characterizing neuroendocrine differentiation in eyelid PCMC remain limited. A 47-year-old man underwent excision of a chalazion-like mass of the right upper eyelid at another clinic, and histopathologic examination revealed mucinous carcinoma. Systemic evaluation found no extracutaneous primary malignancy. Although no residual lesion was clinically apparent at referral, a full-thickness excision approximately 7 mm in total width was performed at the presumed primary site. Histopathologic examination demonstrated residual mucinous carcinoma within the orbicularis muscle, with negative surgical margins. Four months later, firm nodules developed on both sides of the surgical scar, and local recurrence was confirmed. The recurrent lesions were treated with wide full-thickness excision using 7-8 mm clinical margins, and intraoperative frozen-section analysis confirmed negative margins. Staged eyelid reconstruction followed. Immunohistochemistry was negative for synaptophysin and chromogranin A, whereas insulinoma-associated protein 1 (INSM1) showed only focal weak positivity. Gross cystic disease fluid protein-15 (GCDFP-15) was partially positive, while GATA binding protein 3 (GATA3), estrogen receptor, and progesterone receptor were positive. The overall immunophenotype supported apocrine (breast-like) differentiation without significant neuroendocrine differentiation, consistent with non-neuroendocrine PCMC. No recurrence was observed during 36 months of follow-up. This case suggests that histologically negative margins may not exclude occult residual disease when the original tumor site is uncertain. Careful surgical planning, appropriate margin assessment, and long-term surveillance may be warranted because occult residual disease or subclinical tumor spread may contribute to early recurrence.

## Introduction

Primary cutaneous mucinous carcinoma (PCMC) is a rare malignant adnexal neoplasm characterized by epithelial tumor nests floating in abundant extracellular mucin [1,2]. It is a slow-growing tumor that typically affects older adults, and approximately 49.7% of reported cases arise in the periocular region, including the eyelid, canthus, and eyebrow. Clinically, PCMC usually presents as an asymptomatic erythematous nodule [2]. Histologically, it closely resembles metastatic mucin-producing adenocarcinomas of the breast and gastrointestinal tract; therefore, exclusion of an extracutaneous primary malignancy is essential for diagnosis [1]. Local recurrence remains a major clinical concern in eyelid PCMC, with recurrence rates of 30-40% reported after excision without intraoperative margin assessment [3,4].

Recent studies have recognized neuroendocrine differentiation in a subset of PCMC and suggested clinicopathologic and prognostic differences between tumors with and without significant neuroendocrine differentiation [5,6]. Tumors lacking significant neuroendocrine differentiation have been termed non-neuroendocrine PCMC (nnPCMC), and available evidence suggests that they may have a higher risk of local recurrence and metastasis than PCMC associated with neuroendocrine differentiation [5,6]. However, reports of eyelid nnPCMC showing early recurrence despite histologically negative surgical margins remain limited.

We report a Japanese patient with eyelid nnPCMC diagnosed by morphologic and immunohistochemical evaluation. This case is noteworthy because early local recurrence occurred despite histologically negative surgical margins.

## Case presentation

**Figure 1:**
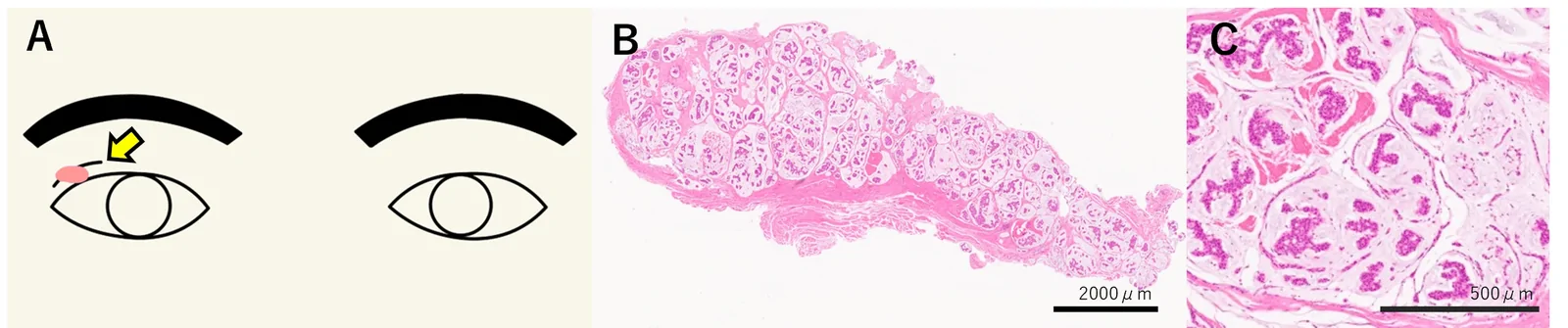
Findings at the referring clinic (A) Schematic illustration of the lesion based on the referral documentation; no preoperative clinical photograph was available. The pink area indicates the presumed location of the original tumor and does not represent its actual color. The lesion measured approximately 3 × 10 mm and was located on the temporal side of the right upper eyelid. The incision site from the initial surgery is indicated by a yellow arrow. Image credit: Panel A is an original schematic created by the authors based on the referral documentation using standard, non-generative drawing tools in Microsoft PowerPoint (Microsoft Corporation, Redmond, Washington, United States). (B) Histopathologic slide from the initial excision provided by the referring clinic, demonstrating features consistent with mucinous carcinoma. Hematoxylin-eosin staining. Scale bar: 2000 µm. (C) Higher magnification view of panel (B), demonstrating the histopathologic features of mucinous carcinoma. Hematoxylin-eosin staining. Scale bar: 500 µm.

A 47-year-old man with no significant past medical or family history presented with a one-year history of swelling of the right upper eyelid. He had initially been treated as having a chalazion, but the lesion did not improve. The following timeline is referenced to his initial presentation to our department. Approximately two months before presentation, a chalazion-like mass on the temporal side of the right upper eyelid was excised through a skin incision at another ophthalmology clinic (Figure 1A). According to the referral documentation, the lesion measured approximately 3 × 10 mm and was described as a fibrous mass with a soft, elastic consistency without adherence to the tarsal plate or surrounding tissues. Its color was not documented, and no preoperative clinical photograph was available. Histopathologic examination of the excised specimen revealed mucinous carcinoma (Figures 1B, 1C), and the patient was subsequently referred to our department for further evaluation and treatment.

**Figure 2:**
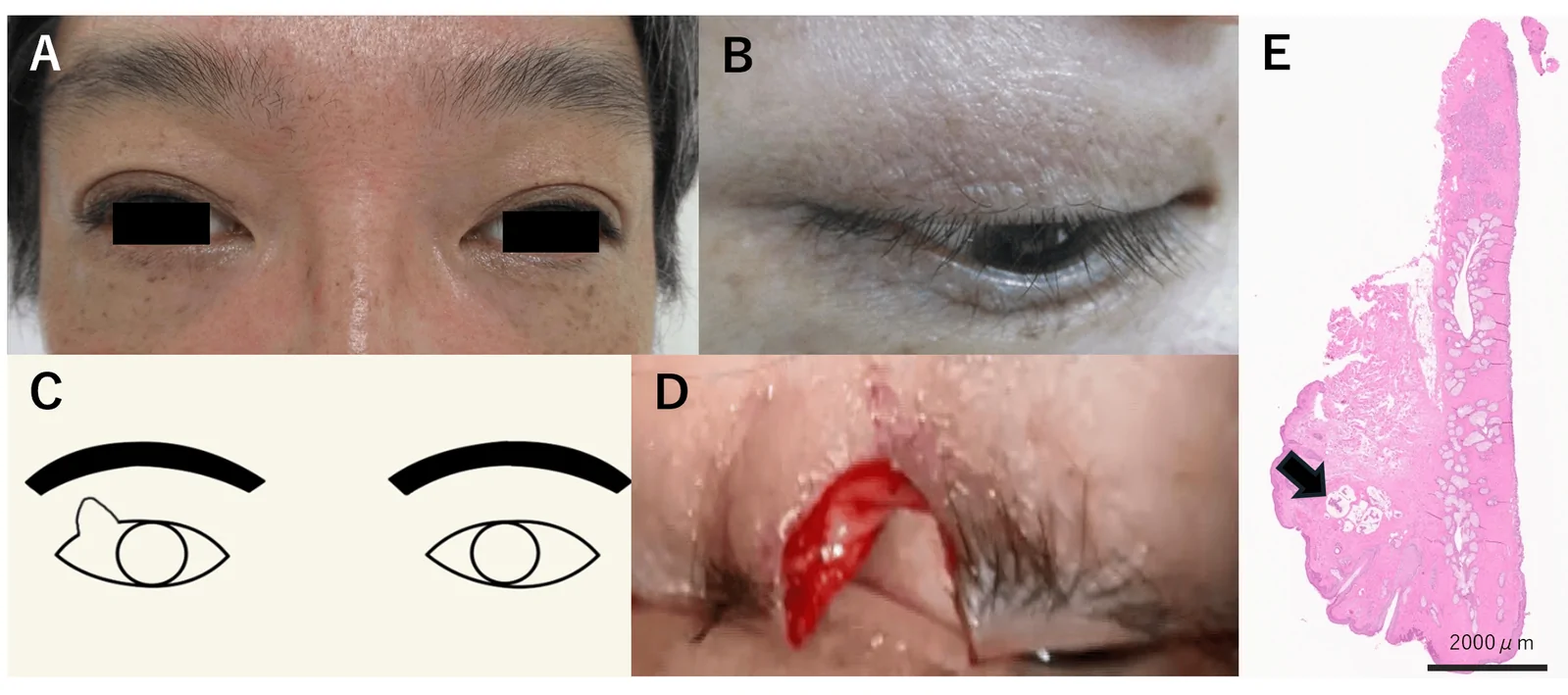
Findings at initial presentation and first additional excision (A, B) Clinical photographs of the right eyelid at presentation. No clinically apparent residual tumor was detected on inspection or palpation. (C, D) Surgical design and intraoperative findings during a full-thickness excision approximately 7 mm in total width, centered on the presumed previous tumor site. Image credit: Panel C is an original schematic created by the authors to illustrate the surgical design using standard, non-generative drawing tools in Microsoft PowerPoint (Microsoft Corporation, Redmond, Washington, United States). (E) Histopathologic findings showing mucin pools and tumor nests within the orbicularis muscle (black arrow). Surgical margins were negative. Hematoxylin-eosin staining. Scale bar: 2000 µm.

At presentation, no clinically apparent residual tumor was detected on inspection or palpation (Figures 2A, 2B). Contrast-enhanced MRI and whole-body PET-CT showed no evidence of regional or distant metastasis. Colonoscopy, breast ultrasonography, and mammography revealed no evidence of an extracutaneous primary mucinous carcinoma, supporting a diagnosis of localized PCMC.

Two months after presentation, additional surgery was performed to evaluate and remove possible residual disease. Because no clinically visible lesion remained, the presumed location of the primary tumor was estimated from the previous surgical scar. A full-thickness excision approximately 7 mm in total width, centered on the presumed previous tumor site, was performed under local anesthesia (Figures 2C, 2D). The 7-mm measurement represented the total width of the excision rather than a 7-mm peripheral margin. This width was selected to permit evaluation and removal of possible residual disease while minimizing the functional and cosmetic impact of the excision on the eyelid. Histopathologic examination revealed tumor nests with mucin pools within the orbicularis muscle, confirming residual mucinous carcinoma; however, all surgical margins were negative (Figure 2E).

**Figure 3:**
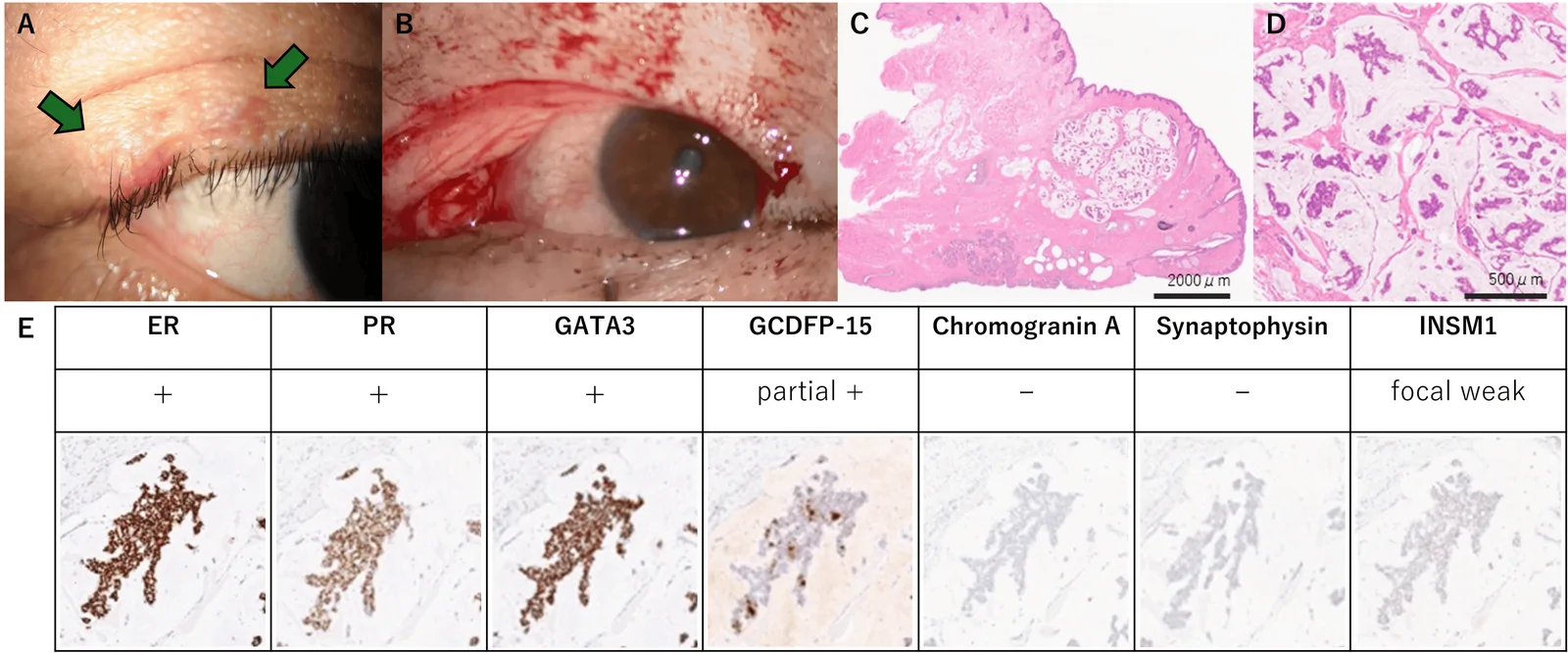
Recurrence, wide re-excision, and histopathologic findings (A) Clinical photograph showing firm nodules on both sides of the previous surgical scar four months after the first additional excision (green arrows). (B) Intraoperative findings during semicircular wide local excision with 7-8 mm margins around the tumor. An artificial dermis was applied to the eyelid defect. (C) Histopathologic findings showing mucinous carcinoma extending from the dermis into the orbicularis muscle. Hematoxylin-eosin staining. Scale bar: 2000 μm. (D) Higher magnification view of panel (C), demonstrating nests and cords of tumor cells floating in abundant extracellular mucin. Hematoxylin-eosin staining. Scale bar: 500 μm. (E) Immunohistochemical findings. Synaptophysin and chromogranin A were negative, whereas INSM1 showed only focal weak positivity. Overall, the morphologic and immunohistochemical findings did not support significant neuroendocrine differentiation. GCDFP-15 was partially positive, whereas GATA3, ER, and PR were positive, supporting apocrine (breast-like) differentiation and a diagnosis of nnPCMC. INSM1, insulinoma-associated protein 1; GCDFP-15, gross cystic disease fluid protein-15; GATA3, GATA binding protein 3; ER, estrogen receptor; PR, progesterone receptor; nnPCMC, non-neuroendocrine primary cutaneous mucinous carcinoma.

Six months after presentation, corresponding to four months after the additional excision, firm nodules developed on both sides of the previous surgical scar (Figure 3A). Two months later, partial excision was performed, and local recurrence of mucinous carcinoma was confirmed histologically.

A further two months later, wide local excision of the upper eyelid was performed under general anesthesia. A semicircular full-thickness excision with 7-8 mm clinical margins around the recurrent lesions was performed (Figure 3B). Intraoperative frozen-section analysis confirmed negative surgical margins. An artificial dermis was applied to the eyelid defect.

Histopathologic examination demonstrated mucinous carcinoma extending from the dermis into the orbicularis muscle, with negative surgical margins (Figures 3C, 3D). Immunohistochemically, synaptophysin and chromogranin A were negative, whereas insulinoma-associated protein 1 (INSM1) showed only focal weak positivity. Gross cystic disease fluid protein-15 (GCDFP-15) was partially positive, whereas GATA binding protein 3 (GATA3), estrogen receptor (ER), and progesterone receptor (PR) were positive (Figure 3E). Overall, the morphologic and immunohistochemical findings did not support significant neuroendocrine differentiation and were consistent with apocrine (breast-like) differentiation and a diagnosis of nnPCMC.

Because the original lesion was excised at another clinic, the orientation of the original specimen was unavailable. Quantitative tumor-to-margin distances and the exact depth of invasion in millimeters were not documented; however, extension into the orbicularis muscle was confirmed.

**Figure 4:**
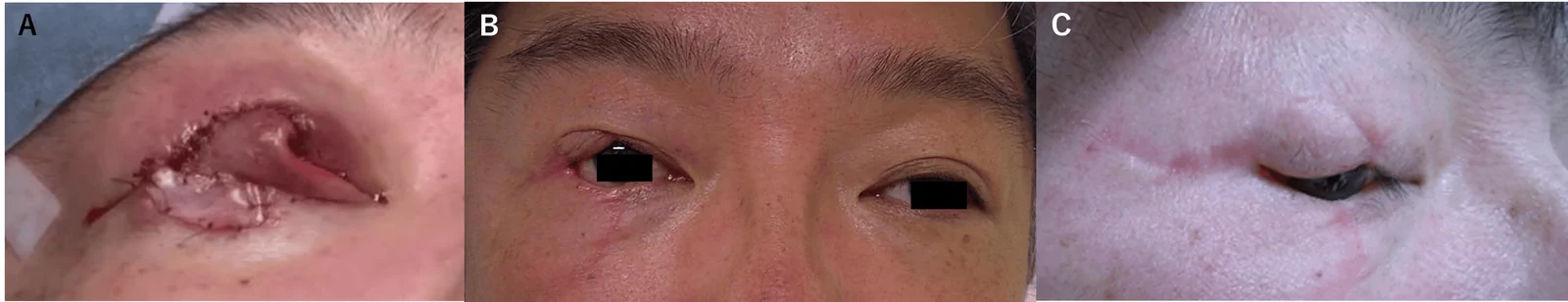
Reconstruction and postoperative outcome (A) Intraoperative photograph of the first-stage eyelid reconstruction performed by the plastic surgery team. A switch flap was elevated from the lower eyelid and transposed to the upper eyelid. (B, C) Clinical photographs obtained 12 months after reconstruction, showing the overall appearance (B) and the right upper eyelid (C). Blinking and eyelid closure were well preserved, with only mild keratoconjunctivitis observed.

Subsequent staged eyelid reconstruction was performed by the plastic surgery team. During the first stage, a switch flap was elevated from the lower eyelid and transposed to the upper eyelid (Figure 4A). During the second stage, the flap was divided and the upper eyelid was reconstructed, followed by reconstruction of the lower-eyelid defect using a malar flap from the right cheek. No local recurrence or clinical evidence of regional or distant metastasis was observed during 36 months of follow-up, and both functional and cosmetic outcomes were satisfactory (Figures 4B, 4C).

The clinical course is summarized in Table 1.

**Table 1:** Clinical timeline of presentation, interventions, histopathologic findings, and follow-up Time points are referenced to the patient’s initial presentation to our department.

Time relative to presentation	Clinical event or intervention	Findings and outcome
Approximately two months before presentation	Excision at another ophthalmology clinic	A 3 × 10 mm chalazion-like mass without adherence to the tarsal plate or surrounding tissues was excised; histopathologic examination revealed mucinous carcinoma
At presentation	Clinical and systemic evaluation	No clinically apparent residual tumor; no extracutaneous primary mucinous carcinoma or regional or distant metastasis identified
Two months after presentation	First additional excision	Full-thickness excision approximately 7 mm in total width; residual carcinoma within the orbicularis muscle; surgical margins negative
Six months after presentation	Clinical recurrence	Firm nodules developed on both sides of the previous surgical scar
Eight months after presentation	Partial excision	Local recurrence of mucinous carcinoma confirmed histologically
Ten months after presentation	Wide local re-excision	Full-thickness excision with 7–8 mm clinical margins; frozen-section analysis confirmed negative margins
After wide local re-excision	Staged eyelid reconstruction	Upper-eyelid reconstruction using a lower-eyelid switch flap and reconstruction of the lower-eyelid defect using a malar flap
Thirty-six months after the final excision	Follow-up	No local recurrence or clinical evidence of regional or distant metastasis; satisfactory functional and cosmetic outcomes

## Discussion

The most notable feature of the present case was the development of local recurrence only four months after the first additional excision despite histologically negative surgical margins. The patient was also relatively young at presentation (47 years), compared with the mean age at diagnosis of 63.5 ± 13.2 years reported by Kamalpour et al. [2]. Because no clinically apparent residual lesion was identified at the time of referral, a full-thickness excision approximately 7 mm in total width was performed at the presumed site of the primary tumor. However, histopathologic examination demonstrated residual mucinous carcinoma within the orbicularis muscle, suggesting that microscopic residual disease may have remained beyond the resected area.

PCMC is characterized by abundant extracellular mucin and scattered tumor nests, making accurate assessment of tumor boundaries difficult. Subclinical tumor spread has been proposed as a possible contributor to local recurrence [3,7]. In the eyelid, adequate surgical margins are often difficult to obtain because of functional and cosmetic constraints. Mohs micrographic surgery has been proposed as a useful treatment option because it allows complete circumferential margin assessment during surgery [4]. However, no consensus has been established regarding the optimal surgical margin for PCMC, with reported margins ranging from 5 mm to an average of approximately 12.5 mm [8,9]. In the present case, no recurrence has been observed during 36 months of follow-up after wide local excision with 7-8 mm margins.

Recent studies have recognized neuroendocrine differentiation in a subset of PCMC and suggested that its presence may be associated with clinicopathologic and prognostic differences [5,6,10]. In the present case, the overall morphologic and immunohistochemical findings did not support significant neuroendocrine differentiation, whereas the expression of apocrine markers supported breast-like differentiation, consistent with nnPCMC. Some studies have suggested that PCMC lacking significant neuroendocrine differentiation may have higher risks of local recurrence and metastasis than endocrine mucin-producing sweat gland carcinoma-associated PCMC with neuroendocrine differentiation [5,6]. In this case, occult residual disease related to uncertainty regarding the original tumor site may have contributed to the early recurrence, although causality cannot be established and a contribution from tumor biology cannot be excluded.

## Conclusions

In conclusion, this case suggests that histologically negative margins in a re-excision specimen may not exclude residual disease outside the sampled area when the original tumor site is uncertain. Occult residual disease or subclinical tumor spread may have contributed to the recurrence; however, causality cannot be established from a single case. Careful surgical planning and long-term follow-up may therefore be appropriate in the management of eyelid PCMC.
